# Advancements in alveolar bone grafting and ridge preservation: a narrative review on materials, techniques, and clinical outcomes

**DOI:** 10.1186/s40902-024-00425-w

**Published:** 2024-04-16

**Authors:** Suyoung Kim, Seong-Gon Kim

**Affiliations:** Department of Oral and Maxillofacial Surgery, College of Dentistry, Gangneung-Wonju National University, Gangneung, 25457 Republic of Korea

**Keywords:** Alveolar ridge preservation, Bone regeneration, Tooth extraction, Bone graft

## Abstract

This narrative review systematically explores the progression of materials and techniques in alveolar ridge preservation (ARP). We commence by delineating the evolution from traditional ARP methods to cutting-edge alternatives, including platelet-rich fibrin, injectable bone repair materials, and hydrogel systems. Critical examination of various studies reveals these innovative approaches not only accelerate bone healing but also significantly improve patient-reported outcomes, such as satisfaction, pain perception, and overall quality of life. Emphasis is placed on the correlation between advanced ARP techniques and enhanced patient comfort and clinical efficacy, underscoring their transformative potential in dental implantology. Highlighting the effectiveness of ARP, the implant survival rate over a span of 5 to 7 years was high, showcasing the reliability and success of these methods. Further, patients expressed high aesthetic satisfaction with the soft tissue outcome, evidenced by an average visual analog scale (VAS) score of 94. This positive aesthetic appraisal is linked to the clinical health of implants, potentially due to the employment of tooth-supported surgical guides. The economic analysis reveals a varied cost range for bone graft substitutes ($46.2 to $140) and socket sealing materials ($12 to $189), with a noteworthy correlation between the investment in barrier membranes and the diminished horizontal and vertical ridge resorption. This suggests that membrane usage significantly contributes to preserving ridge dimensions, offering a cost-effective strategy for enhancing ARP outcomes. In conclusion, this review illuminates the significant advancements in ARP, highlighting the shift towards innovative materials and techniques that not only promise enhanced bone regeneration and reduced healing times but also improve patient satisfaction and aesthetic outcomes. The documented high implant survival rate and the beneficial economic implications of membrane use further validate the effectiveness of contemporary ARP strategies, paving the way for their broader adoption in dental implantology.

## Background

Following tooth extraction, a common physiological consequence is bone resorption due to the loss of physical stimuli to the alveolar bone, which can compromise the structural integrity of the alveolar ridge [1, 2]. This morphological change is not only detrimental to the bone structure but also presents challenges for future dental implant placements [3]. In the context of dental surgery, alveolar ridge preservation (ARP) is aimed specifically at minimizing post-extraction bone loss, thereby preserving both hard and soft tissues at the extraction site. This preservation is crucial for facilitating the successful placement of dental implants in the future [4].

Preservation of the alveolar ridge volume and contour is crucial not only for maintaining healthy and functional dentition but also for averting complex and costly reconstructive surgeries later [5]. By preserving sufficient bone volume, ARP enhances the predictability of dental implant therapy, a critical factor for the stability and longevity of dental implants [6]. Particularly in the anterior maxillary region where esthetics are of prime concern, ARP significantly contributes to favorable aesthetic outcomes [7].

Adequate bone volume and favorable bone morphology are pivotal for successful dental implant placement. By curbing post-extraction bone resorption, ARP preserves these bone characteristics, thereby laying a proper foundation for implant placement [1, 2]. Additionally, by preventing the collapse of soft tissues into the extraction socket, ARP aids in maintaining the gingival architecture essential for natural-looking restorations [4, 7]. Beyond improving implant survival rates, ARP also hastens the healing process allowing for a faster transition to definitive prosthetic replacement, which is advantageous both functionally and for enhancing the patient's self-esteem and satisfaction with the treatment [5, 6].

One contemporary approach to alveolar ridge preservation involves the immediate placement of dental implants into the extraction socket following tooth extraction. However, this technique often encountered challenges in achieving primary stability and managing soft tissue aesthetics due to inadequate bone volume and morphology [1, 2]. Traditional bone graft materials such as autografts, allografts, xenografts, and alloplasts have been utilized to sustain the volume of the alveolar ridge, yet they come with their set of challenges like donor site morbidity, potential disease transmission, and lack of biological interaction [8]. The use of barrier membranes in guided bone regeneration (GBR) to protect the blood clot and segregate the graft material from the overlying soft tissue showed promise, although limitations like membrane exposure, infection, and the need for additional surgical intervention for membrane removal were significant drawbacks [5, 7]. The quest for ideal materials to fill the socket and support new bone growth led to mixed results, with complications such as material migration, infection, and delayed healing.

The advent of biomimetic materials, recent advancements in cellular and molecular therapies, and modern technologies like 3D printing and injectable hydrogels have heralded a new era in ARP [8]. These advancements are better at mimicking the natural bone microenvironment, which holds promise for improved bone regeneration and preservation outcomes [9]. For instance, the incorporation of mesenchymal stem cells and vascular endothelial cells in bone repair materials is a significant leap toward achieving enhanced bone regeneration and vascularization [10]. Innovative approaches like the development of injectable hydrogels that can be photo-cured to stabilize within the socket offer minimally invasive applications that conform to the complex anatomy of the extraction socket [11]. Additionally, modern 3D printing technologies and scaffold designs allow for the creation of patient-specific implants and materials, potentially improving ARP outcomes by offering tailored treatment solutions [6].

This review aims to furnish an analysis of recent studies exploring the efficacy and clinical outcomes of various materials used in ARP, from natural to synthetic, and traditional to novel materials like injectable bone substitutes and 3D-printed scaffolds. An examination of different techniques and surgical procedures in ARP, their evolution over time, and how recent advancements are overcoming the limitations of historical approaches will be provided. Moreover, a critical evaluation of the clinical outcomes associated with different materials and techniques will be discussed, focusing on bone regeneration, implant success, aesthetic outcomes, and patient satisfaction. Lastly, a comparative analysis based on available clinical evidence will be presented to guide clinicians in choosing the most suitable approach for ARP, tailored to individual patient needs and clinical scenarios.

## Main text

### I. Materials employed in ARP

#### A. Bone substitutes

**Fig. 1:**
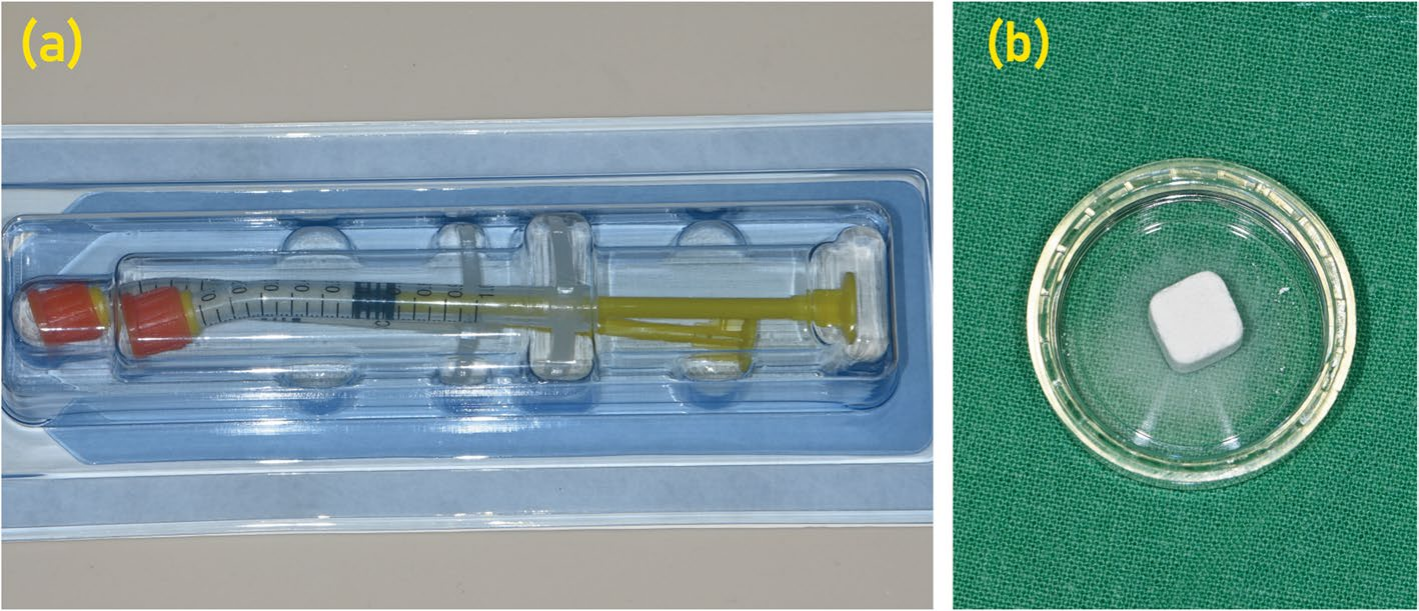
Graft materials. **a** Allografts, (**b**) xenografts from bovine bone

Bone substitutes are graft materials utilized in ARP to facilitate optimal bone regeneration post-tooth extraction. There are three distinct categories allografts, xenografts, and synthetic bone substitutes (Fig. 1). Allografts, derived from human donors and usually procured from cadavers, are processed to ensure safety and efficacy before use [12]. They include types like demineralized bone matrix (DBM) and freeze-dried bone allograft (FDBA), acting as osteoconductive scaffolds to promote bone growth [13]. On the other hand, xenografts are obtained from different species, commonly bovine or porcine, serving as biocompatible scaffolds for bone regeneration, with bovine bone mineral being a prevalent type [14]. They exhibit osteoconductive properties, aiding in the growth and development of the patient's bone cells.

Studies by Wardani et al. [15] and Saliba et al. [16] explored the application of allografts and xenografts respectively in ARP. While Wardani et al. [15] noted favorable outcomes with allografts, Saliba et al. [16] found significant wound healing potential and reasonable bone regeneration with xenografts, albeit with heightened pain perception. The juxtaposition of allografts and xenografts highlights their diverging characteristics. Human-derived allografts may offer better biological integration, whereas bovine-derived xenografts with their longer resorption rates provide a sustained scaffold, albeit potentially delaying complete bone regeneration [12, 14]. While allografts carry a risk of disease transmission and immunogenic reactions, albeit minimal due to stringent processing standards [12], xenografts pose lesser risk owing to the interspecies barrier, albeit with concerns over prion diseases [7]. Both graft types have shown promising clinical outcomes in bone volume preservation and regeneration [15, 16]. However, the heightened pain perception associated with xenografts as noted in the Saliba et al. study [16] necessitates further investigation. Economic factors, availability, regional regulations, and patient preferences may also influence the choice between allografts and xenografts [7].

#### B. Platelet concentrates

**Fig. 2:**
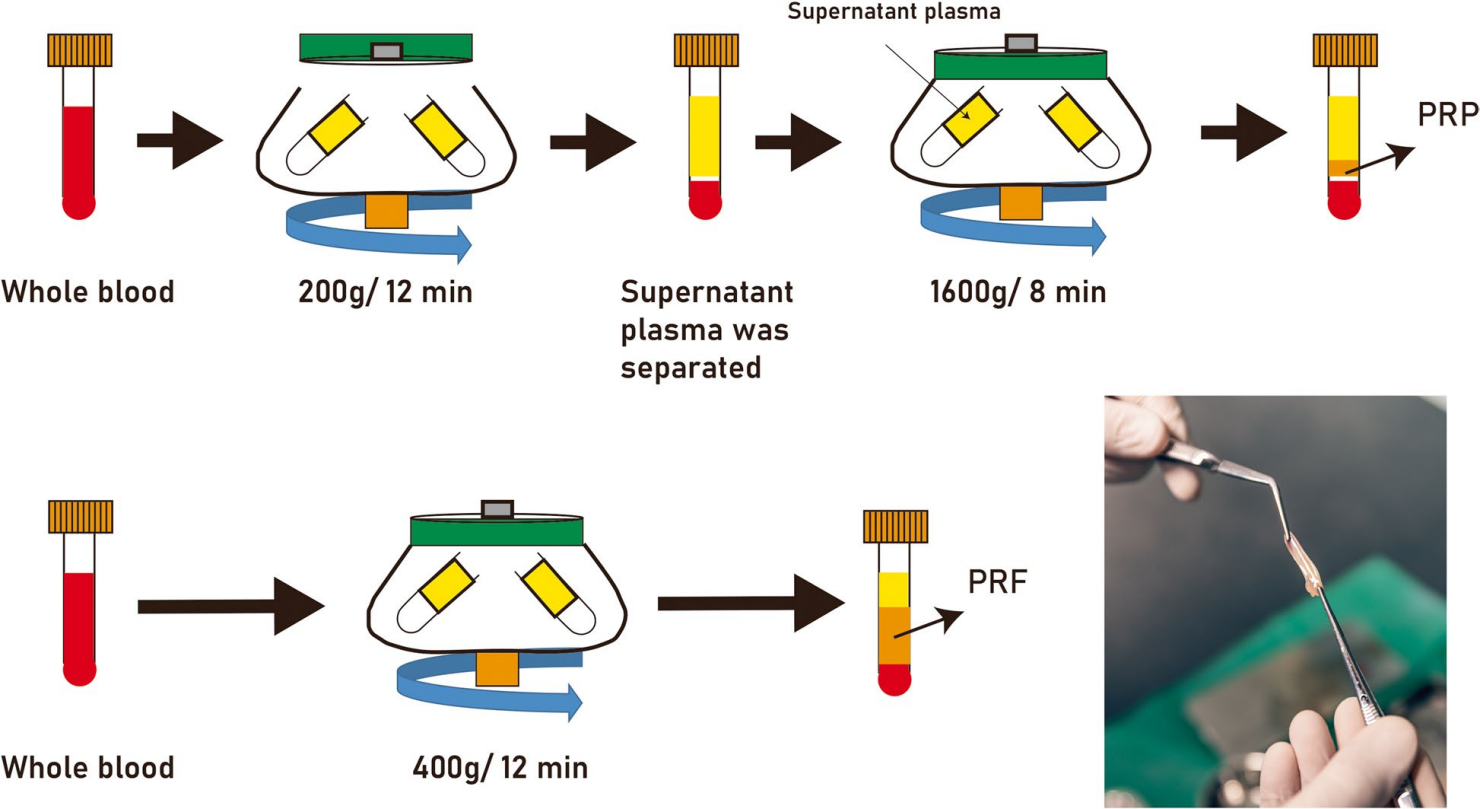
Preparation of platelet-rich plasma (PRP) and platelet-rich fibrin (PRF). PRP is produced through a two-step differential centrifugation process. First, red blood cells (RBCs) are separated during an initial centrifugation phase. Subsequent centrifugation concentrates the platelets, which are then suspended in a minimal plasma volume. This process leverages varying specific gravities to sediment cellular components based on acceleration force adjustments. On the other hand, PRF represents a second-generation autologous platelet concentrate derived from whole venous blood. After a brief centrifugation (~ 10 min) of blood in vacutainer tubes without anti-coagulants, a fibrin gel rich in growth factors, platelets, leukocytes (comprising nearly half of the initial blood sample), and lymphocytes is harvested. This gel is characterized by its slow and strong polymerization

Platelet-rich fibrin (PRF), recognized as a second-generation platelet concentrate hailing from autologous blood, demonstrates significant promise in oral and maxillofacial surgical domains due to its proficient wound healing, angiogenesis, and bone regeneration attributes (Fig. 2). Distinct from its predecessor, platelet-rich plasma (PRP), the absence of anticoagulants in PRF facilitates a slow, natural polymerization sequence during centrifugation (Fig. 2). This process culminates in the creation of a dense fibrin clot enriched with fibrin fibers, platelets, leukocytes, and a plethora of growth factors.

In a recent exploration, Lahham et al. [17] investigated the repercussions of recurrent applications of concentrated PRF (C-PRF) within extraction sockets. The study unveiled notable reductions in hard tissue loss within the test group over a span of 3 months, thereby hinting at C-PRF’s potential in alleviating ridge alterations post-extraction and fostering bone regeneration endeavors [17]. The enriched content of growth factors and bioactive components within PRF notably amplifies socket healing and tissue regeneration. This presents a viable alternative to conventional grafting materials, which necessitate a significantly prolonged integration period before proceeding with implant placements [18].

Upon examining the efficacies of diverse plasma concentrates (PCs) in ARP scenarios, it was ascertained that PCs significantly contribute to new bone formation during ARP in contrast to spontaneous healing [19]. Noteworthy is that among the analyzed PCs, no substantial differences were discerned between leukocyte- and platelet-rich fibrin (L-PRF) and pure platelet-rich plasma (P-PRP), underscoring the potential applicability of either concentrate in ARP instances [19]. Furthermore, Madi et al. [20] substantiated the efficacy of PRF in socket preservation through a methodical review that evaluated the impact of various grafting materials on newly formed bone, both histologically and radiographically. Among the scrutinized materials, PRF emerged prominently for its capability to promote satisfactory new bone formation whilst preserving ridge contour [20]. In another review, Santos Pereira et al. [21] dissected the advantages of advanced platelet-rich fibrin (A-PRF) in tissue regeneration amid reconstructive and jaw graft surgery. The insights from the study positioned A-PRF as a beneficial adjunct in sustaining ridge profile, bolstering bone density, and expediting tissue repair post-extraction [21]. Additionally, the review illuminated the potential of A-PRF in alleviating post-operative pain and swelling, alongside contributing to swifter epithelial healing [21].

The wealth of studies consistently accentuates the auspicious nature of PRF, along with its advanced variant, in enhancing the healing ambiance within oral surgical sites, predominantly post-tooth extractions. Collectively, these findings articulate a persuasive argument for integrating platelet concentrates in clinical regimens to augment bone regeneration and overall tissue healing, thereby smoothening the trajectory for successful dental implant installations.

#### C. Collagen membranes

**Fig. 3:**
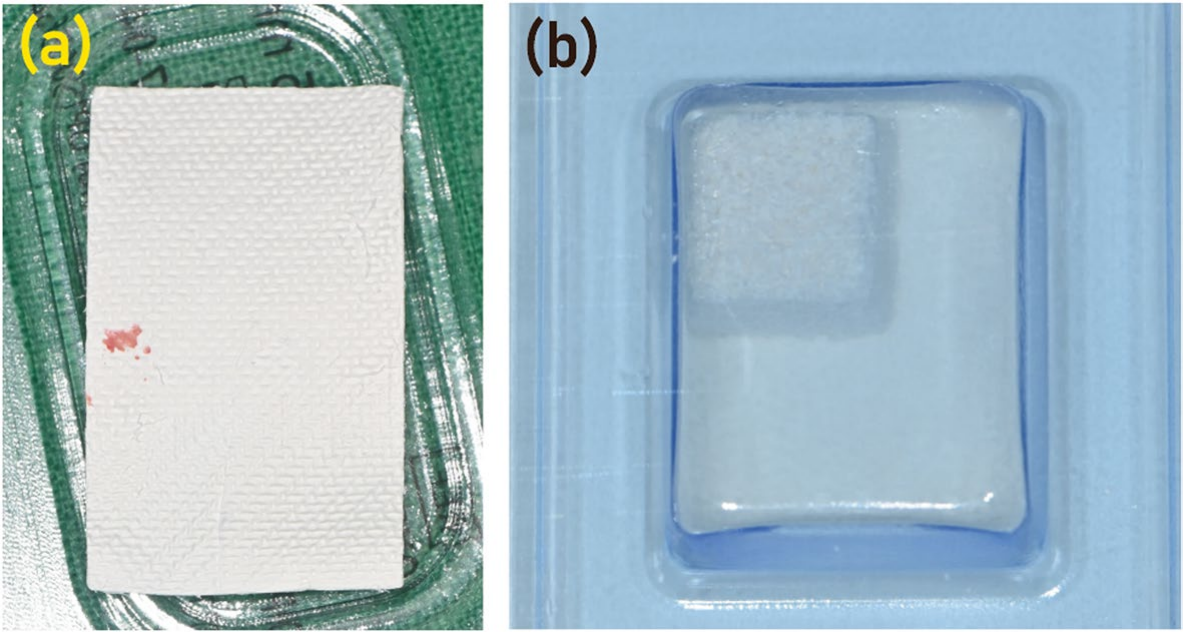
Bone substitutes, such as synthetic grafts and autogenous bone grafts, serve as scaffolds that support the growth of new bone tissue. **a** Synthetic grafts, including hydroxyapatite, tricalcium phosphate, and bioceramics, offer a biocompatible alternative to natural bone grafts. **b** Collagen membranes, derived from various animal sources or obtained through recombinant technology, have gained popularity due to their excellent biocompatibility, biodegradability, and versatile mechanical properties

Collagen membranes are extensively employed with bone substitutes in ARP procedures to reduce bone resorption and promote bone regeneration post-tooth extraction (Fig. 3). Collagen membranes, either derived from animal connective tissues or synthesized, serve as barriers that prevent soft tissue invasion into the bone defect, thereby enabling bone regeneration (Fig. 3A). These membranes are bioresorbable, eliminating the need for secondary surgery for removal, and create a conducive environment for bone healing. Several studies have evaluated the efficacy of bone substitutes and collagen membranes in ARP. For instance, a study highlighted that the deproteinized bovine bone graft and absorbable collagen membrane were beneficial in preserving the alveolar ridge bone, showing no adverse effect on the osseointegration of delayed implants [22]​. Another trial aimed to reduce the dimensional changes in the alveolar bone post-tooth extraction by using an equine collagen membrane and a collagen cone, suggesting the potential of collagen materials in ARP [23].

Different biomaterials have been explored in various studies, including porcine bone substitutes, allografts, alloplasts like biphasic calcium phosphate and β-tricalcium phosphate (Fig. 3B), along with collagen and high-density polytetrafluoroethylene membranes [24]. A systematic review compared the dimensional changes and histological features between anorganic bone and collagen-preserving bone in ridge preservation procedures, promoting awareness of different bone xenograft efficacies in stimulating the healing of post-extraction sockets [2]. Furthermore, a study demonstrated that the use of a bone substitute covered with a collagen membrane resulted in fewer changes in vertical and horizontal alveolar ridge dimensions compared to the collagen membrane alone [25].

A tomographic evaluation by Binkhorst et al. [26] revealed significant preservation of bone volume post-extraction and enhanced bone density when using bone substitutes and collagen membranes, indicative of improved bone quality. This conducive healing period further enabled subsequent restorative procedures like implant placement. While these findings are promising, extended and long-term studies are necessary to ascertain the optimal types and combinations of bone substitutes and collagen membranes for varying clinical scenarios. The utilization of bovine porous bone mineral in conjunction with collagen membrane showed slightly more benefit in preserving alveolar ridge dimensions post-tooth extraction, compared to using bovine porous bone mineral with the autologous fibrinogen/fibronectin system [27]. The diverse findings across different studies underscore the necessity for further research to establish the optimal materials and techniques for ARP, tailored to individual clinical scenarios.

#### D. Bioactive materials

Bioactive materials, encompassing hydroxyapatite (HA), collagen, and three-dimensional (3D) bone repair materials, are at the forefront of advancements in ARP post-tooth extraction. Leveraging modern 3D printing technologies, these materials are engineered to mimic the natural bone structure and composition, thus promoting enhanced integration and regeneration.

A notable exploration in this domain is by Guo et al. [11], who developed a minimally invasive bone repair material. Their innovative design, a 3D bone repair material, comprises a photocurable polyether F127 diacrylate hydrogel loaded with mixed spheroids of mesenchymal stem cells (MSCs) and vascular endothelial cells (ECs). The MSC-EC-F127DA system demonstrated remarkable potential in promoting bone repair and preserving the alveolar ridge shape, marking a significant stride towards effective ARP procedures [11].

Moreover, emerging biomimetic materials, injectable bone substitutes, and patient-specific implants and materials are pivotal in enhancing ARP outcomes. For instance, injectable bone substitutes have been utilized in a range of studies for alveolar bone regeneration and immediate implant placement post-tooth extraction [28, 29]. Particularly, patient-specific titanium mesh has been recognized as a novel approach for stabilizing the augmentation region using particulate bone substitute materials combined with autologous bone, albeit with noted complications like dehiscence [30]. Moreover, the combined use of xenogeneic bone substitute material covered with a native bilayer collagen membrane has shown promise in alveolar ridge preservation, as evidenced by a randomized controlled clinical trial [31].

These advancements underscore a progressive trajectory in ARP, augmenting dental implant success, aesthetic outcomes, and overall patient satisfaction through the integration of bioactive materials. This sphere of bioactive materials not only holds promise in surmounting challenges posed by traditional materials and methods but also signifies an ongoing quest toward optimizing ARP for varying clinical scenarios.

### II. Techniques employed in ARP

#### A. Minimally invasive delivery methods

**Fig. 4:**
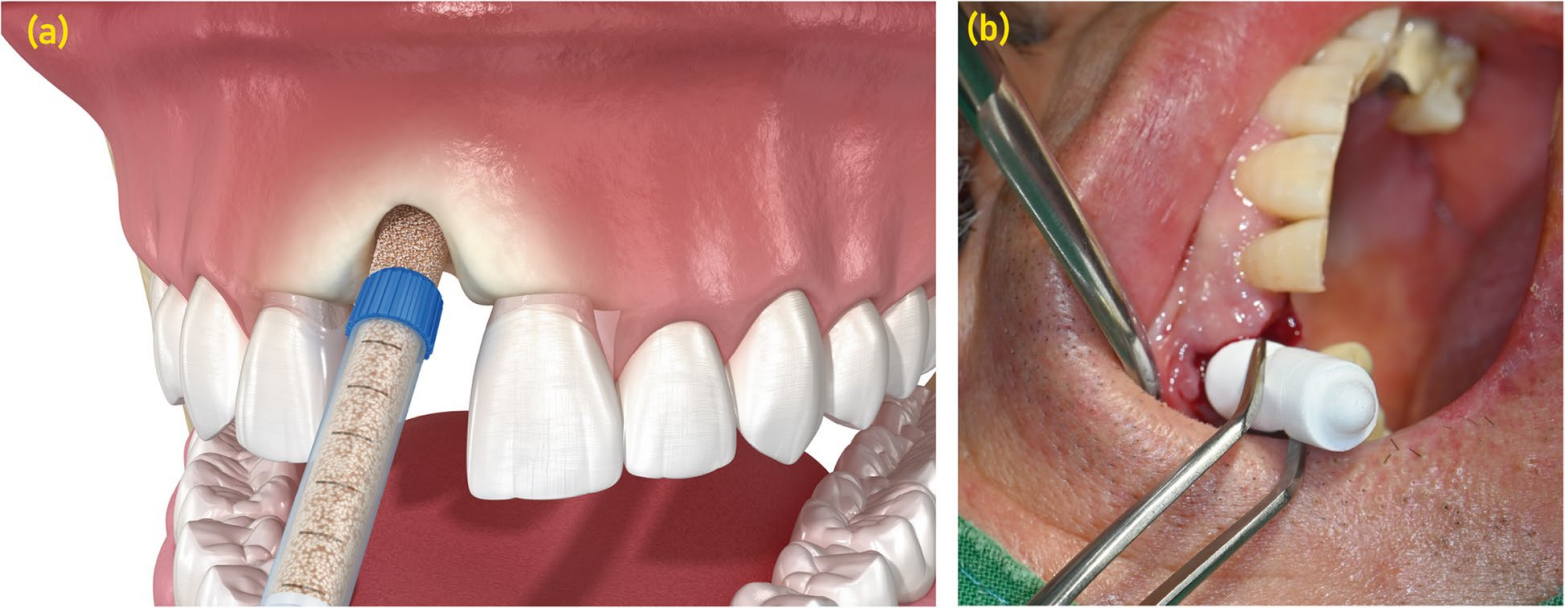
Techniques for socket preservation. **a** Schematic drawing of socket preservation (Image was purchased from Adobe Stock). **b** Socket preservation in the maxillary canine extraction defect focuses on maintaining the dimensions and quality of the extraction socket following tooth removal

Injectable bone repair materials are an emergent avenue in bone regeneration and repair, with applications extending to dental and orthopedic domains (Fig. 4A). These materials are crafted for minimal invasive administration, simplifying the operational aspects, and hastening post-operative recovery. They adapt to the bone defect’s shape upon injection, offering a scaffold for bone regeneration.

Various substances constitute injectable bone repair materials, including natural and synthetic materials such as calcium phosphate cements, bioactive glasses, and hydrogels. Some of these materials are bioresorbable, allowing for the natural bone tissue to replace the scaffold as it degrades over time. The primary objective is to foster a conducive environment for bone cell attachment, proliferation, and eventual regeneration, a crucial aspect of ARP and implant dentistry​ [15, 32].

Guo et al. [11] pioneered an injectable bone repair material that stabilizes upon curing. This material showcased active spreading, filling the defect, and offering a robust scaffold for bone regeneration [11]. Recent advancements have also been seen in other types of injectable materials like ternary calcium-based bone cement, which has been shown to promote bone repair [33], and hierarchically degradable bioactive scaffolds that mimic the natural bone repair process [34]. Furthermore, injectable nanocomposite hydrogels, particularly those based on calcium phosphate and alginate, have also shown promise in supporting angiogenic and osteogenic cell functions, which are crucial for bone repair [35].

These novel materials and the associated minimally invasive delivery methods are transcending the barriers posed by traditional graft materials and procedures, heralding a new era of regenerative therapies in dental and orthopedic applications.

#### B. Comparative analysis of techniques based on the properties of materials

The conventional techniques for ARP and bone regeneration, including the use of allografts, xenografts, and barrier membranes, have been longstanding practices in clinical settings (Fig. 4B). They have been effective to a certain extent in promoting bone regeneration and ensuring successful dental implant placement. However, they pose challenges such as the risk of infection, longer healing times, and sometimes insufficient bone regeneration, which could hinder the success of subsequent dental implant placement [36, 37].

Novel materials and techniques have emerged with the potential to address some of the limitations associated with conventional approaches. Among these novel approaches are the following:

1. Platelet concentrates: studies have proposed that PRF can be used to support bone regeneration during alveolar ridge augmentation. PRF, combined with bone graft materials, might increase bone regeneration, although the efficacy of PRF in enhancing bone regeneration in ARP remains a topic of investigation [38–40].
2. Injectable bone repair materials: as illustrated by the works of Guo et al. [11], injectable bone repair materials provide a minimally invasive approach to bone repair, showcasing significant potential in preserving the alveolar ridge post-extraction.
3. Bioactive materials: these materials, including HA and bioactive glasses, mimic the natural bone structure and composition, fostering better integration and regeneration.

The comparative analyses between conventional and novel techniques have shown varying results. For instance, a systematic review showed that procedures with allografts produced the highest bone percentages at 3 months (54.4%), while those using xenografts yielded the lowest at 5 months (23.6%) [41]. Other studies have compared different ARP techniques such as GBR, socket seal (SS) technique, or unassisted socket healing, revealing radiographic bone changes following ARP [42]. Moreover, some studies have evaluated the combination of different materials and techniques, like combining PRF with different bone graft materials, indicating potential benefits in bone regeneration [39].

Despite the promising outcomes observed with the application of novel techniques and materials, challenges persist. These challenges include the need for standardized protocols, long-term clinical data to establish efficacy and safety, specialized training for clinicians, and understanding the biological mechanisms underlying these novel materials and techniques. The cost considerations, regulatory approvals, and the learning curve associated with the adoption of novel techniques also pose hurdles for widespread acceptance and application in clinical practice.

The field could indeed benefit from multidisciplinary research collaborations encompassing material science, cellular biology, and clinical dentistry. Ongoing and upcoming projects, such as multi-center clinical trials investigating the efficacy of different biomaterials and techniques, hold the potential to further illuminate the path towards optimizing results and improving patient satisfaction in ARP and implant dentistry. Both conventional and novel techniques play pivotal roles in the evolving landscape of ARP and implant dentistry. While conventional techniques provide a reliable foundation, the infusion of novel materials and methods propels the field towards new horizons, fostering a conducive environment for enhanced bone regeneration and successful dental implant therapy.

### III. Clinical and histomorphometric outcomes

#### A. Bone maturity, new bone formation, and resorption rates

The success of ARP hinges significantly on the ability to foster new bone formation, ensure bone maturity, and control bone resorption rates. Here is a more detailed analysis of these factors based on various studies:

1. New bone formation: a study evaluated healing at molar extraction sites and found that using FDBA and an absorbable collagen sponge could effectively preserve the ridge dimension without affecting the amount of new bone formation [43].
2. Bone resorption rates: a 3-year prospective randomized clinical trial aimed to analyze the changes in alveolar bone crest levels and differences in resorption rates between various grafting materials used in ARP post-tooth extraction. The study spanned evaluations over 1, 2, and 3 years of clinical function [44]. Another study observed that alveolar ridge resorption often occurs within the first 6 months post-tooth extraction, with the resorption rate decreasing gradually over the years. This resorption is a crucial factor affecting the longevity and stability of dental implants post-ARP [45].
3. Use of concentrated growth factors: two studies investigated the application of concentrated growth factors (CGFs) in conjunction with other materials for ARP: One study evaluated the effect of CGFs combined with deproteinized bovine bone mineral (DBBM) on ARP during implantology, indicating that this combination might be beneficial for ARP [46]. Another study, a split-mouth, randomized, controlled clinical trial, investigated ARP in post-extraction sockets using concentrated growth factors, hinting at the potential of CGFs in promoting bone preservation and regeneration post-tooth extraction [47].

These studies collectively illustrate the potential of innovative techniques and materials in enhancing bone regeneration, controlling bone resorption, and thus contributing to the success of ARP procedures. However, it is evident that the choice of materials and techniques plays a pivotal role in these outcomes. Further longitudinal and comparative studies could provide more insights into optimizing ARP protocols for improved clinical outcomes.

#### B. Evaluation of pain management and wound healing

In the realm of ARP, managing post-operative pain and evaluating wound healing is crucial for assessing the overall success of the procedures and ensuring patient satisfaction. Various studies have shed light on different aspects of pain management and wound healing following ARP procedures.

One study compared the wound healing potential and pain management efficacy of collagen and xenograft bovine bone covered by a cellulose mesh when inserted into the socket of extracted teeth [16]. Another study discussed the role of maresin 1, a pro-resolving lipid mediator, in accelerating extraction wound healing, promoting socket bone fill, preserving alveolar ridge bone, and reducing post-operative pain, as tested in a rodent preclinical model [48].

Additionally, a randomized controlled trial evaluated the impact of administering hyaluronic acid gel following ARP procedures in terms of changes in wound dimensions over time, which could be a marker for wound healing [49]. A different clinical trial investigated the effectiveness of amnion-chorion membranes in ARP, including clinical, radiologic, and morphometric assessments of wound healing [50]. Furthermore, a histologic study provided evidence regarding vital bone formation and dimensional changes when different types of bone allografts were used in ridge preservation of non-molar tooth sites, which could indirectly relate to wound healing [51]. The research by Saliba et al. [16] demonstrated that collagen supports a quicker wound healing rate, a higher potential influence on socket healing, and a reduction in pain perception when compared to xenograft bovine bone following tooth extraction procedures. This research insight is valuable for assessing post-operative patient comfort and the wound healing process, which are critical for the overall success and patient satisfaction in ARP procedures.

These studies collectively contribute to a growing body of knowledge that underscores the importance of effective pain management and thorough wound healing evaluation in ARP procedures, which can significantly impact the patient’s experience and the success of subsequent dental implant placement.

#### C. Impact on subsequent implant placement and stability

The prime goal of ARP is to create a favorable milieu for the successful placement and stability of dental implants. Various studies have ventured to elucidate the correlation between diverse ARP methodologies and the enduring success of implants, revealing a positive association between efficacious ARP strategies and enhanced implant stability.

The adequacy of bone volume in three dimensions is indispensable for implant osseointegration, making ARP and augmentation crucial for implant therapy [52]. A particular study highlighted that employing particulate xenogenic or allogenic materials, shielded with absorbable collagen membrane or sponge, was linked with favorable outcomes concerning horizontal ridge preservation [2, 16]. It was noted that additional bone augmentation to facilitate implant placement in a prosthetically acceptable position was required in 48.1% of non-grafted extraction sites versus only 11.5% of ARP sites [53]. Interestingly, sites that underwent ARP displayed no difference compared to those that experienced unassisted socket healing regarding implant loss or success [52].

A systematic review aimed to juxtapose the clinical outcomes, including success rates, between delayed implant placement post-ARP and immediate implant placement. While detailed findings are not provided, this review emphasizes the relevance of examining different timelines of implant placement following ARP [54]. A retrospective cohort study involving 108 patients assessed the long-term (5 years) impact of ARP with xenograft bone mineral on peri-implant health, highlighting the potential long-term benefits of ARP on implant stability and overall peri-implant health [55]. A prospective clinical trial explored the impact of ARP and primary stability as influencing factors on the transfer accuracy of static guided implant placement, showcasing the potential implications of ARP on the accuracy and success of implant placement procedures [56].

The amalgam of these studies delineates the multifaceted benefits and significant positive influence ARP possesses on subsequent implant placement and stability [57, 58]. By preserving and augmenting the alveolar bone volume, ARP lays a sturdy foundation for implant placement, substantially mitigating the odds of implant failure, and by extension, bolstering the long-term stability and success of dental implants. This underscores the indispensability of rigorous ARP procedures in enhancing implant stability, which in turn, contributes to the overarching clinical success, and reiterates the essence of ARP in achieving optimal conditions for dental implant placement.

#### D. Long-term effect of ARP

In an analysis involving 288 patients undergoing ARP with immediate implant placement, followed for a period ranging from 3 to 60 months, it was found that 26 of 274 cases (9.5%) experienced complications or adverse effects associated with ARP [59]. However, a substantial majority (90.5%) enjoyed successful implant survival without complications [59]. Another study, a retrospective cohort involving 108 patients, observed that approximately 41% of the 308 implants were placed in sites previously treated with ARP using deproteinized bovine bone mineral xenograft [60]. This study reported a patient-level implant survival rate of 93.7%, with 13 out of 308 implants lost in 10 out of 108 patients [60]. Notably, after 5 years, the patients displayed full-mouth plaque and bleeding scores of 27.7 ± 18.1% and 12.4 ± 12.9%, respectively, alongside an average marginal bone loss of approximately 2.2 mm per patient [60].

Further longitudinal analysis indicated that, although minor hard and soft tissue remodeling occurred within the first 3 months post-ARP, alveolar bone dimensions remained stable from 3 months onwards into the long-term follow-up [61]. This period also witnessed significant improvements in soft tissue profiles at more cervical levels [61]. Remarkably, the implant survival rate after 5 to 7 years reached 100%, with both peri-implant bone levels and soft tissue health being favorably reported [61]. Additional evidence demonstrated the efficacy of ARP in maintaining ridge width and height at the 12-month mark, showing comparability to the 4-month post-procedure outcomes [62]. Such findings suggest that delaying implant placement for up to a year does not detrimentally affect the healed ridge’s height and width, thus providing clinicians with valuable reassurance [62].

Every case under review saw the successful reconstruction of the atrophied alveolar ridge, enabling the placement of intraosseous implants according to the initial treatment plans [63]. Post-treatment, patients were subject to annual monitoring, with the follow-up periods averaging 39 months (spanning 28 to 50 months). During these follow-up intervals, no implants were reported lost [63].

### IV. Patient-centered outcomes

#### A. Patient satisfaction, pain perception, and quality of life post-ARP procedures

The quest for assessing patient satisfaction in the aftermath of ARP procedures can be discerned in various studies and reviews. Notably, a systematic review aimed to juxtapose clinical data, including success rates, tissue preservation, aesthetic results, and patient-reported outcomes between delayed implant placement following ARP and immediate implant placement. This hints at a structured approach toward gauging patient satisfaction through patient-reported outcomes, although the precise method of assessment such as standardized questionnaires or follow-up interviews was not elucidated [54]. In a related study [64], pain and aesthetic satisfaction post-ARP were assessed using visual analogue scales (VAS). At suture removal, the reported pain intensity averaged 17 (SD = 11, range 5–40), indicating minimal discomfort [64]. One-year post-ARP, satisfaction with the aesthetic outcome of the soft tissues was highly rated, with an average VAS score of 94 (SD = 6, range 85–100). This high level of aesthetic satisfaction correlates with the clinical health of implants, potentially attributed to the use of tooth-supported surgical guides for implant installation [64]. Accordingly, the implementation of ARP procedures can be a measure to mitigate post-extraction bone resorption effects, indicating a potential avenue for evaluating patient satisfaction through the assessment of aesthetic and functional outcomes [54].

Another retrospective cohort study explored the efficacy of ARP in reducing the necessity for ridge augmentation at posterior tooth sites, albeit without directly mentioning the assessment of patient satisfaction. The methodologies for this study entailed enrolling patients who underwent dental implants at specified sites between 2013 and 2019, with a collection of demographic data and dental treatment histories, possibly alluding to a patient-centric approach in evaluating the outcomes of ARP procedures [45]. Lastly, a methodological approach to evaluating ARP underscored the aesthetic results of implant restoration, particularly in the anterior maxilla region, and its relation to the soft tissue profile. This study hinted at a paucity of investigations assessing the external soft tissue profile post-ARP procedures, potentially indicating an area where patient satisfaction could be explored further [65].

#### B. Concerning patient discomfort and clinical efficacy

Matumoto et al. [66] conducted a pivotal study aiming to bridge the gap between clinical efficacy and patient discomfort in ARP procedures, shedding light on the correlation between execution and reduced patient discomfort. The study suggested that employing minimally invasive techniques alongside effective pain management strategies could ameliorate post-operative discomfort and expedite the healing process.

Building upon Matumoto et al.’s findings, several other studies have contributed to understanding this interplay [66]. For instance, Couso-Queiruga et al. [67] explored post-extraction dimensional changes, providing insight into the healing process and its impact on patient discomfort. Another study by Wongpairojpanich et al. [68] evaluated the effectiveness of bilayer porous polyethylene membranes for ARP, with findings suggesting the potential to reduce patient discomfort and enhance clinical efficacy.

Moreover, the clinical efficacy of ARP procedures has been substantiated by numerous studies. Avila-Ortiz et al. [69] conducted a systematic review and meta-analysis on the effects of alveolar ridge preservation post-tooth extraction, underlining the significance of ARP in maintaining alveolar ridge dimensions and facilitating optimal conditions for subsequent dental implant placement. Additionally, Couso-Queiruga et al. [70] in a different study, demonstrated that ARP reduces the need for ancillary bone augmentation, thereby enhancing the clinical efficacy of implant therapy. Furthermore, Garcia et al. [71] explored the impact of membrane exposure on guided bone regeneration, contributing to the understanding of potential complications and their management, thereby aligning with the objectives of reducing patient discomfort and ensuring clinical efficacy.

In essence, a confluence of studies reinforces the importance of patient-centered approaches in ARP procedures. By delving into various facets of ARP, from pain management to clinical efficacy, these studies collectively underline the necessity of aligning patient comfort with clinical objectives to drive successful dental implant therapy and enhance patient satisfaction and quality of life.

#### C. Cost-effectiveness ARP

Total cost of standard ARP consists of two main components: the bone grafting material (socket filler) and the barrier (socket sealer), both of which are pivotal in influencing the therapeutic outcomes. Although these components can be mixed in various combinations, certain membrane types are frequently paired with specific bone grafts in both practice and research—for example, porcine collagen membranes with bovine bone graft particles, and dense polytetrafluoroethylene barriers with allograft particles. This diversity implies that no uniform resorption rate can be predicted for a given total expenditure on treatment for an extraction socket. Furthermore, the study speculates on the possibility that the effectiveness of a particular type of membrane might vary when used in conjunction with a specific bone graft type.

The costs for bone graft substitutes and socket sealing materials varied between US$46.2 to US$140 and US$12 to US$189, respectively [72]. A significant correlation was found between the amount spent on barrier membranes and reduced horizontal and vertical ridge resorption, indicating a beneficial effect of membrane use on preserving ridge dimensions [72]. Conversely, the expenditure on bone grafts did not significantly affect ridge resorption either horizontally or vertically. These findings remained consistent even after excluding control sites from the analysis, reinforcing the positive impact of membrane usage on minimizing ridge resorption [72].

Furthermore, the study also included findings from a retrospective study that compared the health-economic and clinical effectiveness of ARP using autogenous bone grafts from the iliac crest (IC) versus demineralized freeze-dried bone (DFDB) before oral implant treatment [73]. This study highlighted that DFDB significantly lowered the costs associated with materials and staff, with the overall treatment cost in the DFDB group being only 22.4% of that in the IC group [73]. Additionally, a similar mixed model was employed to explore the individual contributions of different bone grafts and socket sealing materials to the outcomes, focusing on the nuanced relationships between cost components and clinical effectiveness. The use of allogeneic or xenogeneic bone grafts for alveolar ridge preservation is linked to increased expenses, yet it offers better preservation efficacy compared to employing alloplastic materials or allowing for natural healing [72].

### V. Limitations of the current review

One of the limitations of this narrative review arises from the inherent nature of its design, which does not include specific article selection criteria characteristic of systematic or rapid reviews. Consequently, this approach may contribute to variability in the methodologies, assessment protocols, and outcome measures reported across the included studies, particularly concerning bone regeneration, pain perception, and patient satisfaction in ARP practices.

Furthermore, the review highlights a critical gap in the current literature: the lack of standardized evaluation methods for assessing key outcomes in ARP. The variability in measurement techniques and outcome reporting standards complicates the direct comparison and interpretation of findings across different studies. This inconsistency underscores the necessity for developing and adopting standardized assessment protocols or guidelines within the field. Establishing such guidelines would not only facilitate more accurate comparisons across studies but also enhance the reliability and reproducibility of research findings, contributing to a more robust evidence base for clinical practices in ARP.

As such, while this narrative review provides valuable insights into the existing literature on ARP techniques and materials, the absence of standardized evaluation methods represents a significant limitation. Recommendations for future research include the adoption of uniform assessment protocols to improve the consistency and reliability of study outcomes, thereby advancing our understanding and optimization of ARP procedures.

## Conclusions

In conclusion, the field of ARP has significantly evolved, transitioning from reliance on traditional materials like allografts and xenografts to adopting innovative approaches, including PRF, injectable bone repair materials, and advanced hydrogel systems. This evolution reflects the profound influence of advancements in material science, cellular biology, and 3D printing technologies on dental medicine, addressing previous challenges such as infection risks, prolonged healing times, and suboptimal bone regeneration.

Our comprehensive analysis underscores the remarkable progress in enhancing bone regeneration, reducing recovery durations, and improving clinical outcomes. Future directions for ARP are promising, emphasizing the importance of interdisciplinary collaboration to drive innovation, with a focus on patient-centered outcomes and personalized treatment plans. The integration of novel materials and techniques, alongside the potential of predictive analytics and personalized medicine, holds the potential to revolutionize ARP procedures, making them more effective and tailored to individual patient needs.

As we stand at a pivotal juncture in ARP’s narrative, the continued integration of technological and biological advancements promises to overcome existing limitations, heralding transformative changes in implant dentistry and significantly improving patient care. The collective efforts of the scientific and clinical communities will be crucial in realizing this potential, ensuring that ARP continues to advance in alignment with the overarching goal of enhancing the quality of life for patients.

## Data Availability

Data sharing is not applicable to this article since no dataset was generated or analyzed during the current study.

## Abbreviations

3D: Three-dimensional
A-PRF: Advanced platelet-rich fibrin
ARP: Alveolar ridge preservation
CGFs: Concentrated growth factors
C-PRF: Concentrated platelet-rich fibrin
DBBM: Deproteinized bovine bone mineral
DBM: Demineralized bone matrix
DFDB: Demineralized freeze-dried bone
ECs: Vascular endothelial cells
FDBA: Freeze-dried bone allograft
HA: Hydroxyapatite
IC: Iliac crest
MSCs: Mesenchymal stem cells
PCs: Plasma concentrates
PRF: Platelet-rich fibrin
PRP: Platelet-rich plasma
VAS: Visual analogue scales
